# A Fast, Three-Dimensional, Indirect Geolocation Method Using IAGM and DSM Data without GCPs for Spaceborne SAR Images

**DOI:** 10.3390/s19235062

**Published:** 2019-11-20

**Authors:** Min Liu, Peng Xiao

**Affiliations:** Department of Applied Science and Frontier Technology, Qian Xuesen Laboratory of Space Technology, Beijing 100094, China; xiaopeng@qxslab.cn

**Keywords:** spaceborne synthetic aperture radar (SAR), indirect geolocation, Earth centered rotating (ECR) coordinate system, iterative analytical geolocation method (IAGM), digital surface model (DSM)

## Abstract

To determine the geolocation of a pixel for spaceborne synthetic aperture radar (SAR) images, traditional indirect geolocation methods can cause great computational complexity. In this paper, a fast, three-dimensional, indirect geolocation method without ground control points (GCPs) is presented. First, the Range-Doppler (RD) geolocation model with all the equations in the Earth-centered rotating (ECR) coordinate system is introduced. By using an iterative analytical geolocation method (IAGM), the corner point locations of a quadrangle SAR image on the Earth’s surface are obtained. Then, a three-dimensional (3D) grid can be built by utilizing the digital surface model (DSM) data in this quadrangle. Through the proportional relationship for every pixel in the 3D grid, the azimuth time can be estimated, which is the key to decreasing the calculation time of the Doppler centroid. The results show that the proposed method is about 12 times faster than the traditional method, and that it maintains geolocation accuracy. After acquiring the precise azimuth time, it is easy to obtain the range location. Therefore, the spaceborne SAR image can be geolocated to the Earth surface precisely based on the high-resolution DSM data.

## 1. Introduction

Spaceborne synthetic aperture radar (SAR) images are widely used in a variety of fields [1]. With the paramount need to target surveillance and state security, i.e., target geolocation, the accuracy of the absolute locations of sensitive targets on the ground in spaceborne SAR images is very critical. The absolute accuracy of SAR geolocation depends on multiple factors, such as the orbital precision of the satellite, sensor stability, radar accuracy, knowledge of the propagation medium, SAR processing accuracy, timing accuracy, target detection accuracy, coordinate transformation accuracy, etc. [2,3,4,5,6,7].

The traditional indirect geolocation method establishes the mapping association between image coordinates and geodetic coordinates by repetitively calculating the relation between the digital elevation model (DEM) grid and the satellite position, which requires several processing iterations [8,9,10]. A good deal of work has been done using the traditional indirect geolocation method to achieve good accuracy. Geolocation accuracy can be effectively improved by using the precise latitude, longitude, and elevation provided by ground control points (GCPs), such as Wettzell corner reflector experiment. As reported in [11,12], TerraSAR-X has the capability of submeter-level geolocation accuracy in range. An in-depth analysis of TSX-1 carried out as part of the Wettzell corner reflector experiment, the pixel geolocation accuracy was improved to approximately 1 cm in range [11]. However, especially in overseas, mountainous areas, canyons, deserts, etc., it is difficult to obtain GCPs, while the geolocation of these areas is very important. Therefore, a method of geolocation without GCPs is necessary in such cases. The RD model is the most widely-used physical sensor model for spaceborne SAR, which can be used to geolocate without GCPs [13]. In [14], a fast geocoding method of spaceborne SAR images using graphics processing units was proposed. This method accelerated the algorithm processing by performing block processing and parallel processing using the graphic processing unit or the central processing unit. However, this method is only an engineering implementation, and does not improve the calculation model. In [15], a hybrid algorithm based on the RD location model was proposed, which could achieve a higher level of accuracy than the analytic geolocation method (AGM) and relative geolocation method. This method took 10.9 s to process the first, middle, and last rows of a SAR image [15].

In this paper, a method to improve the Doppler centroid calculation model is proposed by estimating the geometric relationship between a target point inside a SAR image and the four corner points of the quadrangle SAR image. (For a $N_{a}\times N_{r}$ pixels SAR image, the four corner points are located at (1,1), $\left(1,N_{r}\right)$, $\left(N_{a},1\right)$ and $\left(N_{a},N_{r}\right)$. The target point means any pixel except for the four corner points in the $N_{a}\times N_{r}$ SAR image.) This geolocation method for SAR images does not use GCPs, and since the iterative calculations of the Doppler centroid are omitted, it is more efficient to geolocate targets using this method while ensuring the geolocation accuracy. Furthermore, the proposed geolocation method is a general method that can be applied for all current spaceborne SAR systems. In this paper, we choose TanDEM-X images as the experimental data; the experimental results demonstrate the efficiency and reliability of the proposed method.

The rest of the paper is structured as follows. In Section 2 geolocation theory is introduced and divided into four subsections: geometric analyses for SAR images, the RD model for SAR images, the details about the IAGM, and the atmospheric propagation delay for microwaves that contains ionospheric and tropospheric delays. A fast, three-dimensional, indirect geolocation method is then proposed in Section 3. The experimental results in Section 4 validate the performance of the proposed method. Section 5 discusses the results and future works. Finally, Section 6 concludes the findings and contributions of this paper.

## 2. The Geolocation Theory

### 2.1. The Coordinate System and Transformation Formula

**Figure 1:**
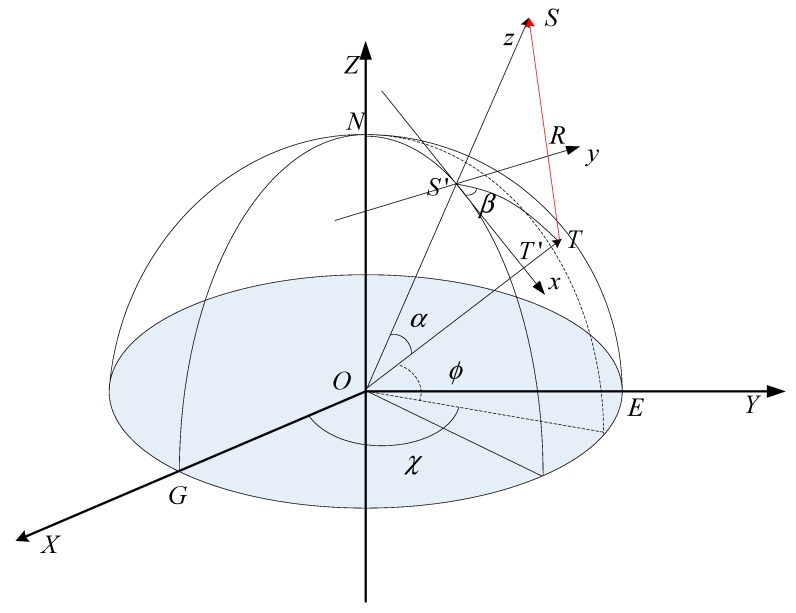
The local coordinate system $S^{\prime }-xyz$ based on ECR coordinate system.

Figure 1 illustrates the local coordinate system $S^{\prime }-xyz$ based on the ECR coordinate system. The ECR coordinate system is widely used for RD model geolocation [16]. As shown in Figure 1, O−XYZ is a geocentric coordinate system, N represents the North Pole, and the arc NG is the Greenwich meridian. O is set as the origin, which coincides with the Earth’s center of mass. The *Z* axis and the mean rotational axis of the Earth coincide; the X axis is pointing to the mean Greenwich meridian, and the Y axis is directed to complete a right-handed Cartesian coordinate system. E is the point where the Y axis passes through the surface of the Earth. $S^{\prime }-xyz$ is the local coordinate system based on the ECR coordinate system. S represents the position of the satellite, and $S^{\prime }$ is the sub-satellite point. T is the target on the Earth’s surface, $T^{\prime }$ is the projected point of T on the ellipsoid surface, $T^{\prime }T$ is the elevation of the target T, and R is the slant range between the satellite and the ground target. $S^{\prime }N$ is an arc passing $S^{\prime }$. $S^{\prime }$ is set as the origin of the local coordinate system. The OS vector is regarded as the *z* axis; the direction of the OS vector is set as the positive direction. The *x* axis is in the ${OS}^{\prime }N$ plane, which passes the subsatellite point $S^{\prime }$ and is perpendicular to line $S^{\prime }S$. The *y* axis is then defined according to the right-handed rule of the Cartesian coordinate system. In addition, α is the angle for ∠TOS, and β is the angle between the plane ${OS}^{\prime }N$ and the plane ${TOS}^{\prime }$. χ is the geocentric longitude of T, and ϕ is the geocentric latitude of T [16,17,18,19,20,21].

In Figure 1, the target T’s location is $\left(x_{T},y_{T},z_{T}\right)$. Its geocentric longitude and latitude in the spherical coordinate system are given by [22]:

(1) $$\tan\chi_{T}=\frac{y_{T}}{x_{T}}$$

(2) $$\tan\phi_{T}=\frac{z_{T}}{\sqrt{x_{T}^{2}+y_{T}^{2}}}$$

in which $\chi_{T}$ is the geocentric longitude of T, and $\phi_{T}$ is the geocentric latitude of T.

In the ellipsoidal coordinate system, the target T’s geodetic longitude and latitude are $\gamma_{T}$ and $\varphi_{T}$, respectively. The target T’s geodetic longitude and geodetic latitude are the same [19]:

(3) $$\tan\gamma_{T}=\frac{y_{T}}{x_{T}}$$

The relation between geocentric latitude and geodetic latitude could be given by [22]:

(4) $$\tan\varphi_{T}=\left(1-e_{c}^{2}\frac{N}{N+h_{T}}\right)\tan\phi_{T}$$

where $e_{c}$ is the first eccentricities of Earth, $h_{T}$ is the ellipsoid height of T. N is the radius of curvature in the prime vertical [19,22]:

(5) $$N=\frac{a}{\sqrt{1-e_{c}^{2}\sin^{2}\varphi_{T}}}$$

$\left(\gamma_{T},\varphi_{T},h_{T}\right)$ are the parameters in the geodetic coordinate system, and $\left(x_{T},y_{T},z_{T}\right)$ are the parameters in the space rectangular coordinate system. The transformations from the geodetic coordinate to the space rectangular coordinate are given by [19,22]:

(6) $$\left[\begin{array}{l} x_{T}\\ y_{T}\\ z_{T} \end{array}\right]=\left[\begin{array}{l} \left(N+h_{T}\right)\cos\varphi_{T}\cos\gamma_{T}\\ \left(N+h_{T}\right)\cos\varphi_{T}\sin\gamma_{T}\\ \left(N\cdot (1-e_{c}^{2})+h_{T}\right)\sin\varphi_{T} \end{array}\right]$$

### 2.2. The RD Model for SAR Images

#### 2.2.1. The Earth Model Equation

Since the Earth’s shape is technically an oblate ellipsoid, the difference between its semimajor and semiminor axes is small. But for precise measurements, we must consider the model of the Earth as an oblate ellipsoid. Therefore, the Earth model equation is given by [23,24]:

(7) $$\frac{x_{T}^{2}+y_{T}^{2}}{a^{2}}+\frac{z_{T}^{2}}{b^{2}}=1$$

where a is the semimajor axis and b is the semiminor axis.

Considering the ellipsoid height, the Earth model equation can be rewritten as [25]:

(8) $$\frac{x_{T}^{2}+y_{T}^{2}}{{\left(a+h_{T}\right)}^{2}}+\frac{z_{T}^{2}}{{\left(b+h_{T}\right)}^{2}}=1$$

#### 2.2.2. The SAR Doppler Equation

The Doppler frequency in the ECR coordinate system for a generic point target is given by [23]:

(9) $$f_{D}=-\frac{2}{\lambda R}\left(\vec{V_{S}}-\vec{V_{T}}\right)\left(\vec{R_{S}}-\vec{R_{T}}\right)$$

where $f_{D}$ is the Doppler frequency, λ is the radar wavelength, $\vec{R_{S}}=\left(x_{S},y_{S},z_{S}\right)$ is the satellite position vector, $\vec{R_{T}}=\left(x_{T},y_{T},z_{T}\right)$ is the ground target position vector, $\vec{V_{S}}=\left({\dot{x}}_{S},{\dot{y}}_{S},{\dot{z}}_{S}\right)$ is the satellite velocity vector, and $\vec{V_{T}}$ is the ground target velocity vector. The velocity vector $\vec{V_{T}}$ in the ECR coordinate system is given by [23]:

(10) $$\vec{V_{T}}=[0,0,0]$$

#### 2.2.3. The SAR Range Equation

The slant range between the sensor and the ground target at given time $t_{i}$ is written as the following expression [23,24,25]:

(11) $$R(i,j)=\left|\vec{R_{S}}-\vec{R_{T}}\right|$$

where $R(i,j)=\sqrt{{\left(x_{S}-x_{T}\right)}^{2}+{\left(y_{S}-y_{T}\right)}^{2}+{\left(z_{S}-z_{T}\right)}^{2}}$, i represents the row number for the SAR image, and j represents the column number for the SAR image. j can be written as [25]:

(12) $$j=\left(R-R_{\min}\right)/\left(\frac{c}{2f_{s}}\right)+1$$

where $R_{\min}$ is the nearest slant range for the SAR image, $f_{s}$ is the sampling frequency, and c is the speed of light.

#### 2.2.4. The RD Model

The RD model is established between a satellite and a ground target on the Earth’s surface [24], which includes Equations (8), (9), and (11). At every azimuth time, the position and velocity vectors of the satellite can be fitted though GPS ephemeris. Therefore, $\vec{R_{S}}$, $\vec{V_{S}}$, and $\vec{V_{T}}$ are known, and the ground target $\vec{R_{T}}$ is unknown, which can be calculated by solving the three equations using AGM.

### 2.3. Iterative Analytical Geolocation Method (IAGM)

As we know, the shape of the Earth is an ellipsoid. Point $T^{\prime }$ is a small area around the subsatellite point $S^{\prime }$, so ${OS}^{\prime }={OT}^{\prime }=R_{L}$. $R_{L}$ is called the local radius, which is given by the following expression [16]:

(13) $$R_{L}=\sqrt{\frac{a^{2}b^{2}}{a^{2}\sin^{2}\phi_{S}+b^{2}\cos^{2}\phi_{S}}}$$

where $\phi_{S}$ is the geocentric latitude of $S^{\prime }$.

According to the cosine theorem, the angle α is given by [16]:

(14) $$\cos\alpha =\frac{E^{2}+F^{2}-G^{2}}{2EF}$$

where $E=R_{S}=\left|\vec{R_{S}}\right|=\sqrt{x_{S}^{2}+y_{S}^{2}+z_{S}^{2}}$, $F=R_{L}+h_{T}$, G=R(i,j). $R_{S}$ is the magnitude of the sensor position vector.

The detailed steps of the IAGM are as follows:

(a) Calculate the geocentric latitude and longitude of the sub-satellite point $S^{\prime }$:
  (15) $$\chi_{S}=\tan^{-1}\left(\frac{y_{S}}{x_{S}}\right)$$
  (16) $$\phi_{S}=\tan^{-1}\left(\frac{z_{S}}{\sqrt{x_{S}^{2}+y_{S}^{2}}}\right)$$
(b) In the local coordinate system, the vectors $\vec{R_{S}}$, $\vec{V_{S}}$, and $\vec{R_{T}}$ in the ECR coordinate system can be transformed to $\vec{R_{Sl}}$, $\vec{V_{Sl}}$, and $\vec{R_{Tl}}$ [16]:
  (17) $$\vec{R_{Sl}}=\left(0,0,z_{Sl}\right)$$
  (18) $$\vec{V_{Sl}}=\left({\dot{x}}_{Sl},{\dot{y}}_{Sl},{\dot{z}}_{Sl}\right)$$
  (19) $$\vec{R_{Tl}}=\left(x_{Tl},y_{Tl},z_{Tl}\right)$$
  where $z_{Sl}=R_{S}-R_{L}$.
  (20) $$\left[\begin{array}{l} {\dot{x}}_{Sl}\\ {\dot{y}}_{Sl}\\ {\dot{z}}_{Sl} \end{array}\right]=\left[\begin{array}{l} -{\dot{x}}_{S}\sin\phi_{S}\cos\chi_{S}-{\dot{y}}_{S}\sin\phi_{S}\sin\chi_{S}+{\dot{z}}_{S}\cos\phi_{S}\\ {\dot{x}}_{S}\sin\chi_{S}-{\dot{y}}_{S}\cos\chi_{S}\\ {\dot{x}}_{S}\cos\phi_{S}\cos\chi_{S}+{\dot{y}}_{S}\cos\phi_{S}\sin\chi_{S}+{\dot{z}}_{S}\sin\phi_{S} \end{array}\right]$$
  (21) $$\left[\begin{array}{l} x_{Tl}\\ y_{Tl}\\ z_{Tl} \end{array}\right]=\left[\begin{array}{l} (R_{L}+h_{T})\sin\alpha \cdot \cos\beta \\ (R_{L}+h_{T})\sin\alpha \cdot \sin\beta \\ -(R_{L}+h_{T})(1-\cos\alpha ) \end{array}\right]$$

Equations (17)–(19) are the parameters for the Doppler equation in the local coordinate system $S^{\prime }-xyz$.

(c) Calculate the angle β from the Doppler equation.

In the local coordinate system $S^{\prime }-xyz$, the Doppler equation is determined by:

(22) $$f_{d}=-\frac{2}{\lambda R}\left(\vec{R_{Sl}}-\vec{R_{Tl}}\right)\cdot \vec{V_{Sl}}$$

Equations (17)–(19) can be directly substituted into Equation (22):

(23) $$f_{d}=-\frac{2}{\lambda R}({\dot{x}}_{Sl}(-(R_{L}+h_{T})\sin\alpha \cos\beta )+{\dot{y}}_{Sl}(-(R_{L}+h_{T})\sin\alpha \sin\beta )+{\dot{z}}_{Sl}(z_{Sl}+(R_{L}+h_{T})(1-\cos\alpha )))$$

where $R=\left|\vec{R_{Sl}}-\vec{R_{Tl}}\right|$.

Suppose [16]:

(24) $$\left\{ \begin{array}{l} A=\frac{2}{\lambda R}{\dot{x}}_{Sl}(R_{L}+h_{T})\sin\alpha \\ B=\frac{2}{\lambda R}{\dot{y}}_{Sl}(R_{L}+h_{T})\sin\alpha \\ C=-\frac{2}{\lambda R}{\dot{z}}_{Sl}(R_{S}+h_{T}-(R_{L}+h_{T})\cos\alpha )-f_{d} \end{array}\right.$$

Therefore, Equation (23) can be rewritten by [16]:

(25) Acosβ+Bsinβ+C=0

The above equation can be solved as [16]:

(26) $$\cos\beta =\frac{-AC\pm B\sqrt{A^{2}+B^{2}-C^{2}}}{A^{2}+B^{2}}$$

The sign “±” depends on the SAR observation mode. If the look direction is right, “+” is chosen. Otherwise, “−” is chosen.

(d) Calculate the geocentric latitude and longitude for the ground point T by using the angle α and the angle β [16].
  (27) $$\cos\left(\frac{\pi }{2}-\phi_{T}\right)=\cos\alpha \cdot \sin\phi_{S}+\sin\alpha \cdot \cos\phi_{S}\cdot \cos\beta$$
  (28) $$\cos(\chi_{S}-\chi_{T})=\frac{\cos\alpha -\sin\phi_{S}\cdot \sin\phi_{T}}{\cos\phi_{S}\cdot \cos\phi_{T}}$$

Transform geocentric latitude and longitude to geodetic latitude and longitude [19,22]:

(29) $$\left\{ \begin{array}{l} \gamma_{T}=\chi_{T}\\ \varphi_{T}=\tan^{-1}\left(\left(1-e_{c}^{2}\frac{N}{N+h_{T}}\right)\tan\phi_{T}\right) \end{array}\right.$$

Therefore, the vector of the ground target is $\vec{R_{T}}=\left(\gamma_{T},\varphi_{T},h_{T}\right)$.

The iterative steps for this AGM are executed in order to obtain more precise result. The process steps are described as follows:

1. Get $R_{L}$, $R_{S}$, and R(i,j) according to the above method.
2. Use Equation (14) to calculate the angle α.
3. According to the steps from (a) to (d), the position vector $\vec{R_{T}}=\left(\gamma_{T},\varphi_{T},h_{T}\right)$ can be obtained.
4. Calculate the value $R_{L}^{\prime }=\left|\vec{R_{T}}\right|-h_{T}$. If $\left|R_{L}^{\prime }-R_{L}\right|<0.01$, then stop the iteration and get the final result $\left(\gamma_{T},\varphi_{T},h_{T}\right)$. Otherwise, let $R_{L}=R_{L}^{\prime }$ and re-execute step 2.

### 2.4. Atmospheric Propagation Delay for Microwaves

The SAR signals travel through the atmosphere slower than they travel in a vacuum due to air refractivity. The distance ΔL is related to the atmospheric refractive index n(s), and can be written as [26]:

(30) ΔL=MF(θ)⋅ΔZ

where ΔZ is the zenith delay, and MF(θ) is the projection function. Generally, the incidence angle of θ for a SAR system is between $20^{{}^{\circ}}$ and $55^{{}^{\circ}}$. A simple projection function is given by [26]:

(31) $$MF(\theta )=\frac{1}{\cos\theta }$$

The zenith delay includes two delay parts: the tropospheric delay and the ionospheric delay [26,27]:

(32) $$\Delta Z=\Delta Z_{tropo}+\Delta Z_{iono}$$

#### 2.4.1. The Ionospheric Delay

The ionospheric refractive index is given by [28]:

(33) $$n_{ph}^{iono}=1-\frac{C_{X}}{2}N_{e}f^{-2}\pm \frac{C_{X}C_{Y}}{2}N_{e}B_{0}f^{-3}-\frac{C_{X}^{2}}{8}N_{e}^{2}f^{-4}$$

where $N_{e}$ is the electron density, $B_{0}$ is the magnitude of the magnetic field vector $B_{0}$, f is the radar frequency in Hz, and the two constants $C_{X}$ and $C_{Y}$ are given by [28]:

(34) $$C_{X}\equiv \frac{e^{2}}{4\pi^{2}\varepsilon_{0}m_{e}}=80.62$$

(35) $$C_{Y}\equiv \frac{\mu_{0}e}{2\pi m_{e}}$$

where e is the electron charge $\left(e=1.602\times 10^{19}C\right)$, $\varepsilon_{0}$ is the permittivity of free space $\left(\varepsilon_{0}=8.854\times 10^{-12}F/m\right)$, $m_{e}$ is the electron mass $\left(m_{e}=9.109\times 10^{-31}kg\right)$, $\mu_{0}$ is the permeability of free space $\left(\;\mu_{0}=4\pi \times 10^{-7}H/m\right)$.

The first two terms in Equation (33) are regarded as the first order refractive index. Due to the much smaller magnitude of the third- and fourth-order terms, only the electron density within the ionosphere is considered [28]. So, Equation (33) can be rewritten as [28]:

(36) $$n_{ph}^{iono}=1-\frac{C_{X}}{2}f^{-2}=1-\frac{40.31N_{e}}{f^{2}}$$

Therefore, the group refractive index is expressed as [28]:

(37) $$n_{gr}^{iono}=1+\frac{C_{X}}{2}N_{e}f^{-2}=1+\frac{40.31N_{e}}{f^{2}}$$

The ionospheric delay can be calculated by the total number of electrons on the propagation path [26]:

(38) $$\Delta Z_{iono}=\frac{40.31}{f^{2}}TEC$$

where TEC is the total electron content.

Therefore:

(39) $$\Delta L_{iono}=MF(\theta )\cdot \Delta Z_{iono}=\frac{40.31}{f^{2}\cos\theta }TEC$$

The radar signal ionospheric delay $\Delta L_{iono}$ converts the path delay from nadir to the path at a constant incidence angle θ.

The real TEC value can be downloaded from the ftp server of the international Global Navigation Satellite Systems (GNSS) service (IGS) [29].

#### 2.4.2. The Tropospheric Delay

Since most SAR systems operate at frequencies below 40 GHz, and the refractivity using the millimeter-wave propagation model (MPM) is more or less constant, the refractivity can be considered frequency independent [5].

In the integral zenith model, the tropospheric delay is divided into hydrostatic, wet, and liquid components [30,31]:

(40) $$\Delta Z_{tropo}=10^{-6}\int_{s}Nds$$

where s is the actual propagation path.

(41) $$N=k_{1}\frac{P_{d}}{T}+k_{2}\frac{e_{w}}{T}+k_{3}\frac{e_{w}}{T^{2}}+1.45\cdot W_{cl}$$

where $k_{1}=0.776KPa^{-1}$, $k_{2}=0.716KPa^{-1}$, and $k_{3}=3.75\times 10^{3}K^{2}Pa^{-1}$ are the constants, $P_{d}$ is the partial pressure of dry air in Pa, $e_{w}$ is the partial pressure of water vapor in Pa, T is the absolute temperature in Kelvin, and $W_{cl}$ is the cloud water content in g/m^3^. The liquid delay is very small, so the last term of liquid water in the above equation can be neglected [7].

The tropospheric delay can be rewritten as [7]:

(42) $$\Delta L_{tropo}=MF(\theta )\cdot \Delta Z_{tropo}=\frac{10^{-6}}{\cos\theta }\int_{z_{0}}^{z_{t}}(k_{1}\frac{P_{d}}{T}+k_{2}\frac{e_{w}}{T}+k_{3}\frac{e_{w}}{T^{2}})ds$$

where $z_{0}$ is the height of the Earth surface, $z_{t}$ is the height of the upper limit of the troposphere.

#### 2.4.3. Real Data for the Ionosphere and Troposphere

The international GNSS service provides orbits, ionospheric delay, tropospheric delay, and other high-quality GNSS data products online in near real time for the whole world [32,33,34,35].

**Figure 2:**
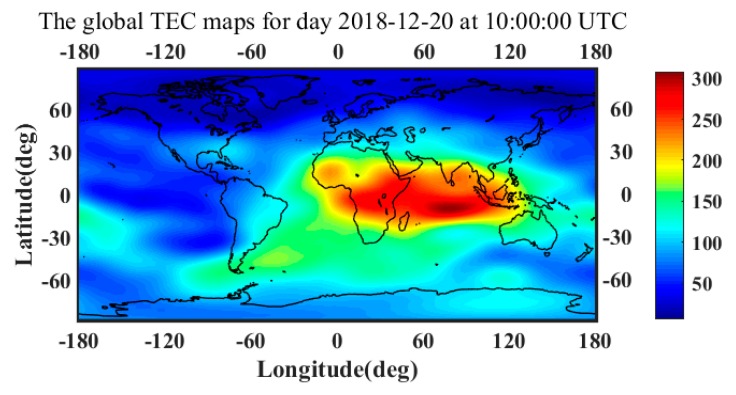
The global TEC maps for day 20 December 2018 at 10:00:00 UTC.

In order to define a global ionospheric model, the vertical total electron content is represented as a function of geocentric longitude, latitude, and time in the form of a raster grid. The global ionosphere maps are provided in the IONospheric EXchange (IONEX) format [29]. The time resolution of the maps is two hours, and the spatial resolution of each gird is 5 degrees in longitude and 2.5 degrees in latitude. Figure 2 shows the global TEC maps at 10:00:00 UTC on 20 December 2018.

**Figure 3:**
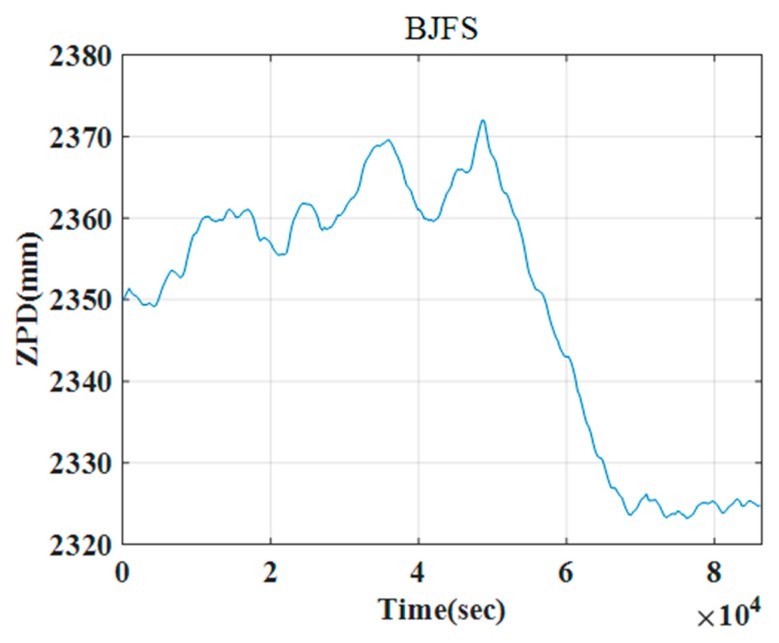
The zenith path delay (ZPD) at the BJFS IGS station on 20 December 2018.

Since 1997, IGS has provided zenith path delay (ZPD) products, which are archived and can be accessed through the FTP server [36]. As shown in Figure 3, the ZPD products for 20 December 2018 are available at Beijing Fangshan (BJFS) station. Therefore, the BJFS data can be used to represent the ZPD of Beijing.

## 3. A Fast, Three-Dimensional, Indirect Geolocation Method

Geodetic coordinates, i.e., latitude, longitude, and height, are required by the traditional indirect geolocation method. Therefore, high precision DEM data or DSM data is the basic requirement for precise geolocation. In short, the Doppler centroid and slant range can be obtained by calculating the relationship between the target geodetic coordinates and the satellite position. Then, the pixel location in the azimuth and range can be obtained according to the Doppler centroid and slant range, respectively. The traditional indirect geolocation method requires iterative calculations to obtain the azimuth time, which is very time consuming. Therefore, in this paper, we introduce a fast indirect geolocation method to speed up the calculation of the azimuth time.

**Figure 4:**
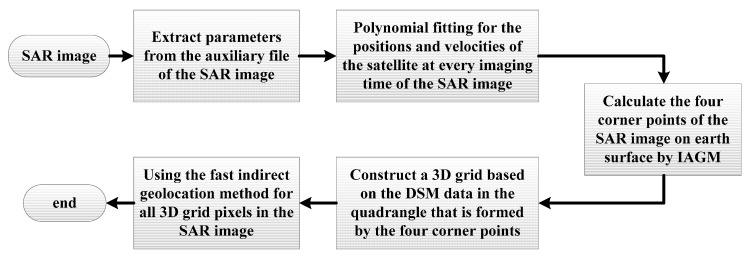
The algorithm process flowchart.

**Figure 5:**
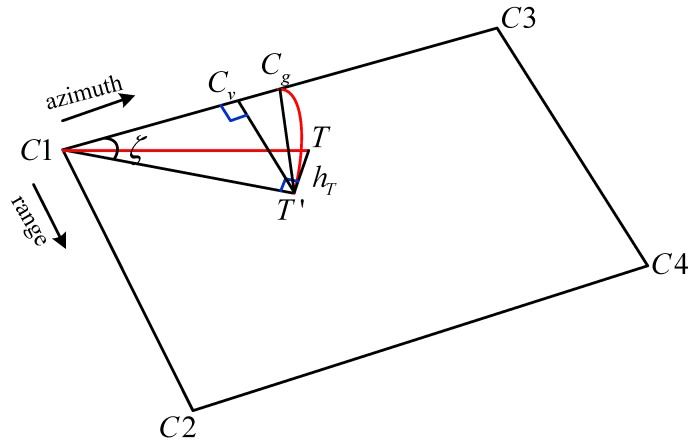
The geometric illustration of the estimated azimuth time.

In order to clearly express the proposed algorithm and the process steps, a flowchart is presented in Figure 4. As shown in Figure 5, the geometric illustration of the estimated azimuth time is given.

Suppose the elevations are zero for the four corner points, which are marked C1, C2, C3, and C4. The corner point C1 refers to the pixel (1,1) in the SAR image, whose geolocation point on the Earth’s surface is $\left(\gamma_{C1},\varphi_{C1},0\right)$. The corner point C2 refers to the pixel $\left(1,N_{r}\right)$ in the SAR image, whose geolocation point on the Earth’s surface is $\left(\gamma_{C2},\varphi_{C2},0\right)$. The corner point C3 refers to the pixel $\left(N_{a},1\right)$ in the SAR image, whose geolocation point on the Earth’s surface is $\left(\gamma_{C3},\varphi_{C3},0\right)$. The corner point C4 refers to the pixel $\left(N_{a},N_{r}\right)$ in the SAR image, whose geolocation point on the Earth’s surface is $\left(\gamma_{C4},\varphi_{C4},0\right)$. The rectangular space coordinate for the four corner points according to Equations (5) and (6) are recorded as $\left(x_{C1},y_{C1},z_{C1}\right)$, $\left(x_{C2},y_{C2},z_{C2}\right)$, $\left(x_{C3},y_{C3},z_{C3}\right)$, $\left(x_{C4},y_{C4},z_{C4}\right)$.

The detailed steps of the proposed fast, three-dimensional, indirect geolocation method are as follows:

(a) Calculate the slopes of the latitude and the longitude between the first azimuth time and the last azimuth time at the nearest and furthest slant ranges, respectively.
  (43) $$K_{L1}=\frac{\gamma_{C3}-\gamma_{C1}}{N_{a}-1}$$
  (44) $$K_{B1}=\frac{\varphi_{C3}-\varphi_{C1}}{N_{a}-1}$$
  (45) $$K_{LN_{r}}=\frac{\gamma_{C4}-\gamma_{C2}}{N_{a}-1}$$
  (46) $$K_{BN_{r}}=\frac{\varphi_{C4}-\varphi_{C2}}{N_{a}-1}$$
  Then, the average values for the slopes of latitude and the longitude are given by:
  (47) $$K_{L}=\frac{K_{L1}+K_{LN_{r}}}{2}$$
  (48) $$K_{B}=\frac{K_{B1}+K_{BN_{r}}}{2}$$
(b) Calculate the distance between C1 and C3:
  (49) $$R_{C1C3}=\sqrt{{\left(x_{C1}-x_{C3}\right)}^{2}+{\left(y_{C1}-y_{C3}\right)}^{2}+{\left(z_{C1}-z_{C3}\right)}^{2}}$$
(c) As point T’s projection point is $T^{\prime }$, the positions of T and $T^{\prime }$ are $\left(\gamma_{T},\varphi_{T},h_{T}\right)$ and $\left(\gamma_{T^{\prime }},\varphi_{T^{\prime }},0\right)$, respectively. The rectangular space coordinates of point T and point $T^{\prime }$ are $\left(x_{T},y_{T},z_{T}\right)$ and $\left(x_{T^{\prime }},y_{T^{\prime }},z_{T^{\prime }}\right)$, respectively. Then calculate the distance between C1 and $T^{\prime }$.
  (50) $$R_{C1T^{\prime }}=\sqrt{{\left(x_{C1}-x_{T^{\prime }}\right)}^{2}+{\left(y_{C1}-y_{T^{\prime }}\right)}^{2}+{\left(z_{C1}-z_{T^{\prime }}\right)}^{2}}$$
(d) Estimate point $C_{g}$ on the straight line C1C3, whose geodetic coordinate $\left(\gamma_{C_{g}},\varphi_{C_{g}},0\right)$ is given by:
  (51) $$\gamma_{C_{g}}=\gamma_{C1}+K_{L}\frac{R_{{C1T}^{\prime }}}{R_{C1C3}}\left(N_{a}-1\right)$$
  (52) $$\varphi_{C_{g}}=\varphi_{C1}+K_{B}\frac{R_{C1T^{\prime }}}{R_{C1C3}}\left(N_{a}-1\right)$$

The rectangular space coordinate for point $C_{g}$ is $\left(x_{C_{g}},y_{C_{g}},z_{C_{g}}\right)$. Therefore, $R_{T^{\prime }C_{g}}$ and $R_{C1C_{g}}$ can be expressed as:

(53) $$R_{T^{\prime }C_{g}}=\sqrt{{\left(x_{T^{\prime }}-x_{C_{g}}\right)}^{2}+{\left(y_{T^{\prime }}-y_{C_{g}}\right)}^{2}+{\left(z_{T^{\prime }}-z_{C_{g}}\right)}^{2}}$$

(54) $$R_{C1C_{g}}=\sqrt{{\left(x_{C1}-x_{C_{g}}\right)}^{2}+{\left(y_{C1}-y_{C_{g}}\right)}^{2}+{\left(z_{C1}-z_{C_{g}}\right)}^{2}}$$

According to the cosine theorem, the angle ζ can be given:

(55) $$\zeta =\cos^{-1}\frac{R_{C1Cg}^{2}+R_{C1T^{\prime }}^{2}-R_{T^{\prime }Cg}^{2}}{2R_{C1Cg}R_{C1T^{\prime }}}$$

Estimate the projection point $C_{v}$ for point $T^{\prime }$ on the straight line $C1C_{g}$.

(56) $$R_{C1C_{v}}=R_{{C1T}^{\prime }}\cdot \cos\zeta$$

(e) From the ratio relationship, the azimuth time $t_{i\text{\_}est}$ of point T can be derived:
  (57) $$t_{i\text{\_}est}=t_{1}+\mathrm{int}(\frac{R_{C1C_{v}}}{R_{C1C3}}\cdot N_{a})\cdot t_{line}$$
  where $t_{1}$ is the SAR image generation starting time, $t_{line}$ is the imaging time interval of the SAR image, and int(⋅) returns the integer part of a decimal number.

According to the geometry estimation, the estimated azimuth time $t_{i\text{\_}est}$ of point T can be obtained, though it has an error that needs to be corrected.

(f) Obtain point T’s precise azimuth time $t_{i}$. Calculate the Doppler frequency at the estimated azimuth time $t_{i\text{\_}est}$ by Equations (9)–(11).
  (58) $$f_{D}(t_{i\text{\_}est})=-\frac{2}{\lambda R}\left(\vec{V_{s}}(t_{i\text{\_}est})\right)\left(\vec{R_{s}}(t_{i\text{\_}est})-\vec{R_{T}}\right)$$
  where $\vec{V_{s}}(t_{i\text{\_}est})=({\dot{x}}_{s}(t_{i\text{\_}est}),{\dot{y}}_{s}(t_{i\text{\_}est}),{\dot{z}}_{s}(t_{i\text{\_}est}))$, $\vec{R_{s}}(t_{i\text{\_}est})=(x_{s}(t_{i\text{\_}est}),y_{s}(t_{i\text{\_}est}),z_{s}(t_{i\text{\_}est}))$, $\vec{R_{T}}=\left(x_{T},y_{T},z_{T}\right)$, $R=\left|\vec{R_{s}}(t_{i\text{\_}est})-\vec{R_{T}}\right|$.

Similarly, we could obtain the Doppler frequencies at the first and last azimuth times for the point at the scene center. The Doppler frequency slope can be written as:

(59) $$K_{f}=\frac{f_{D\text{\_}cent}(t_{N_{a}})-f_{D\text{\_}cent}(t_{1})}{\left(N_{a}-1\right)}$$

Therefore, the precise azimuth time $t_{i}$ can be obtained by the second estimation.

(60) $$t_{i}=t_{i\text{\_}est}-\mathrm{int}\left(\frac{f_{D}(t_{i\text{\_}est})}{K_{f}}+0.5\right)\cdot t_{line}$$

(g) Calculate the slant range of point T.
  (61) $$R=\sqrt{{\left(x_{T}-x_{S\text{\_}t_{i}}\right)}^{2}+{\left(y_{T}-y_{S\text{\_}t_{i}}\right)}^{2}+{\left(z_{T}-z_{S\text{\_}t_{i}}\right)}^{2}}$$
  (62) $$j=\mathrm{int}\left(\frac{R-R_{\min}}{\rho_{r}}+0.5\right)$$
  where $\left(x_{S\text{\_}t_{i}},y_{S\text{\_}t_{i}},z_{S\text{\_}t_{i}}\right)$ is the satellite position at the azimuth time $t_{i}$, $\rho_{r}$ is the range spacing.

The proposed fast, three-dimensional, indirect geolocation method contains seven steps (a~g). By calculating the spatial geometric relationship between the four corner points and point T, the estimated azimuth time $t_{i\text{\_}est}$ can be obtained. Then, the precise azimuth time $t_{i}$ can be easily derived by Equations (58)–(60), and the pixel location in azimuth i is acquired. Finally, by calculating the slant range of point T, the pixel location in range j can be obtained.

## 4. Experiments and Analyses

### 4.1. Geolocation Results

The proposed algorithm is tested with the TanDEM-X’s spotlight mode SAR image. All the parameters are listed in Table 1. The semimajor and semiminor axes are 6378137 m and 6356752.315 m, respectively. The speed of light is 2.99792458E + 5 km/s.

First, we find the relevant DSM data according to the TanDEM-X SAR image, and ensure the gridded DSM is suitable to the resolution of the image. Then, all the steps are executed according to the algorithm process flowchart in Figure 4.

**Figure 6:**
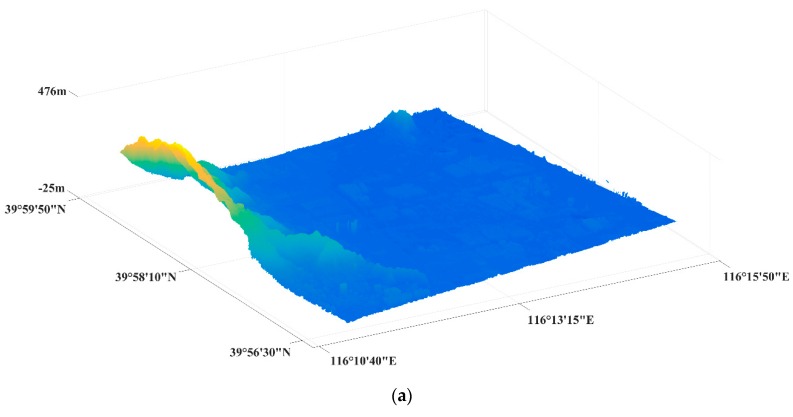

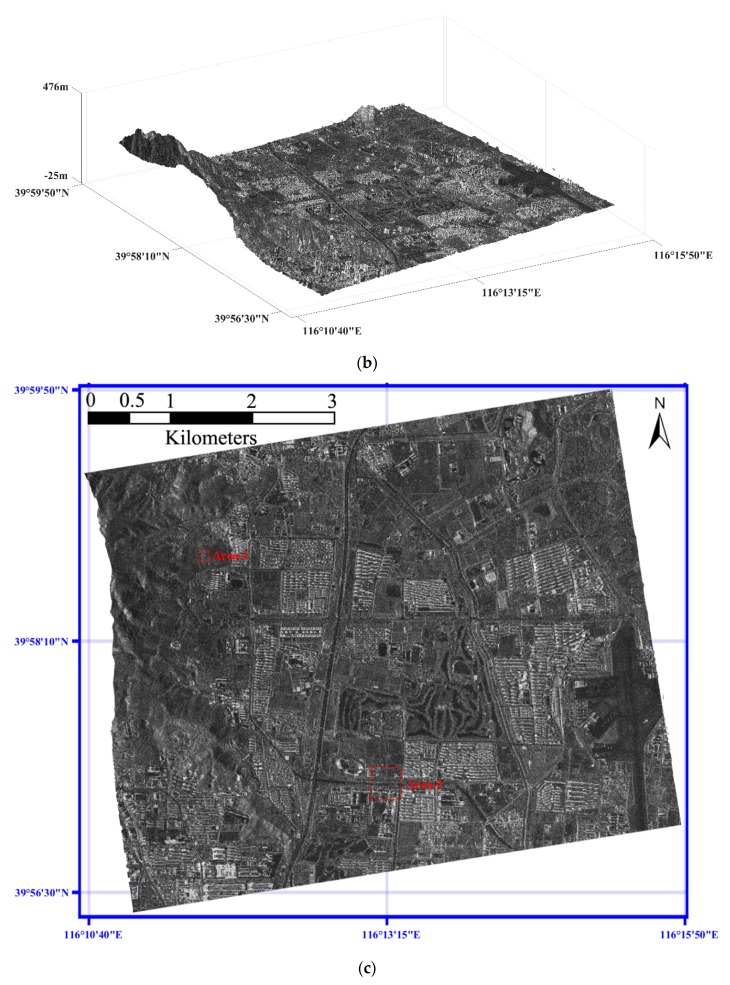
The TanDEM-X SAR image geolocation results (M2). (**a**) DSM. (**b**) The three-dimensional image. (**c**) The two-dimensional image.

Figure 6 shows the TanDEM-X SAR image geolocation results using the proposed algorithm in this paper. This figure gives the geolocation results in geodetic coordinates. Figure 6a provides the 0.69 m × 0.53 m gridded DSM, whose accuracy is the same as elevation 1 [37]. It should be noted that the height of DSM data is orthometric height [38]. The proposed algorithm is deduced based on an ellipsoidal Earth model, which means that the height is ellipsoid height. The geoid height is the difference between the ellipsoid and geoid [38]. Therefore, we assume the ellipsoid height, the orthometric height, and the geoid height are recorded as ‘h’, ‘H’, and ‘N’, respectively. Considering that this experimental area is small, a geoid height at the scene center can replace the other geoid heights in the scene. The geoid height can be easily obtained through the EGM2008 Geopotential Model [39]. The three- and the two-dimensional images are given in Figure 6b,c, respectively. In Figure 6b, the geodetic latitude, geodetic longitude, and ellipsoid height are included for every pixel.

Since the Qianxun Spatial Intelligence Inc. (Qianxun SI, Shanghai, China) offers a positioning service with centimeter-level accuracy (FindCM) for its customers, in this paper, the QianXun (QX) FindCM positioning results are used as the true positions. Two methods are used to compare the algorithm accuracy. Method 1 (M1) has no atmospheric propagation delay correction. Method 2 (M2) has atmospheric propagation delay correction, which includes tropospheric delay and ionospheric delay corrections. From Figure 2, it can be observed that the TEC map in Beijing is about 7.8 TECU. The value of 7.8 TECU is essentially extracted from the IONEX file [29]. Using TEC and other parameters in Equation (39), the ionospheric delay of the TanDEM-X SAR image can be obtained. Figure 3 shows that the ZPD of Beijing area is 2.368 m. Using Equation (42), the tropospheric delay can be acquired. The proposed method can be easily proved in M1 and M2, with M2 obviously being a better choice than M1.

**Figure 7:**
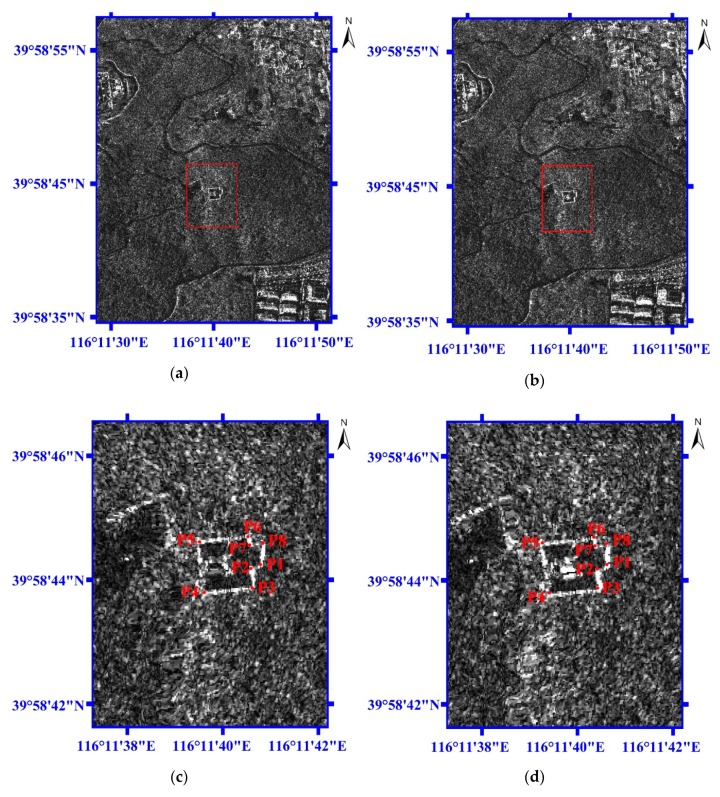
The geolocation results for Banshan Pavilion at Western Hills in Beijing. (**a**) The two-dimensional image for M1. (**b**) The two-dimensional image for M2. (**c**) The enlarged image for the red window (Area 1 in Figure 6) in Figure 7a. (**d**) The enlarged image for the red window (Area 1 in Figure 6) in Figure 7b.

Figure 7 presents the geolocation results for the Banshan Pavilion at Western Hills in Beijing which is picked up from Figure 6. The image size of Figure 7 is 1024 × 1024 pixels. Figure 7a,b are the two-dimensional images for M1 and M2, respectively. In order to see the eight check points (P1~P8) at the Banshan Pavilion clearly, the red windows in Figure 7a,b are enlarged and shown in Figure 7c,d, respectively. The red windows in Figure 7a,b have the same longitude and latitude ranges. Compared with Figure 7c, the eight check points (P1~P8) at Banshan Pavilion in Figure 7d obviously shift several pixels longitudinally.

**Figure 8:**
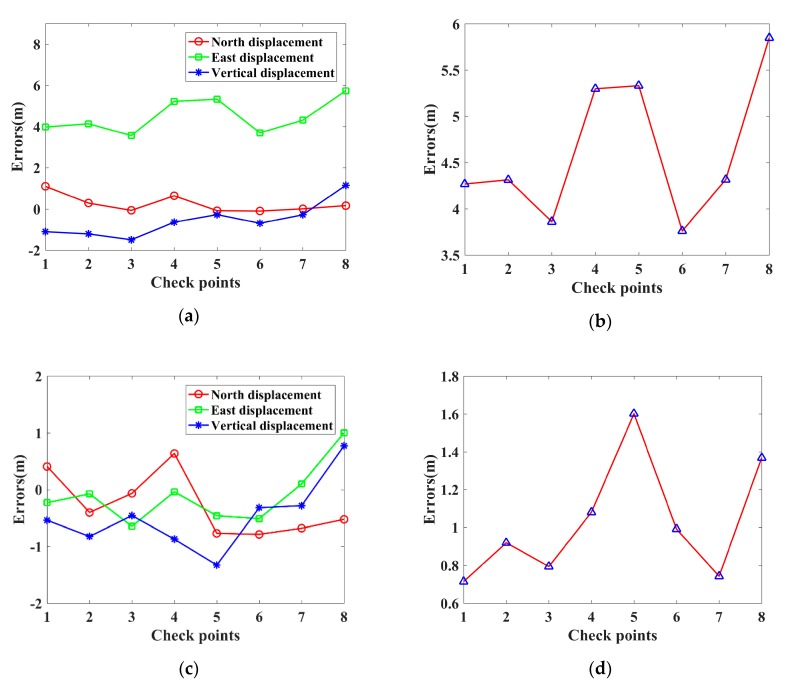
The geolocation errors for the Banshan Pavilion at Western Hills in Beijing with two methods. (**a**,**c**) are the geolocation errors in the north, east and vertical displacements for M1 and M2, respectively. (**b**,**d**) are the three-dimensional errors for M1 and M2, respectively.

Figure 8 illustrates the geolocation errors for the Banshan Pavilion with these two methods. Figure 8a,c illustrate the geolocation errors in the north, east, and vertical displacements for M1 and M2, respectively. The red line with circle markers represents the north displacement, the green line with rectangle markers represents the east displacement, and the blue line with asterisks represents the vertical displacement. In Figure 8b,d, the red lines with blue triangle markers represent the three-dimensional errors for M1 and M2, respectively.

Table 2 presents the geolocation accuracy for the eight corner points. The QX positioning results are taken as the true positions for the eight check points. By comparing the geolocation results of M1 (or M2) with QX, we can get the geolocation accuracy of the check points. As shown in Table 2, there are large displacements in east for M1, which are mainly due to atmospheric propagation delay in range. The displacements of the eight corner points in northing (ΔN), easting (ΔE), orthometric height (ΔH), and spatial (Δ***Spatial***) are summarized in Table 2. The M1 geolocation errors are very large; the three-dimensional errors range from 3.76 m to 5.85 m. The M2 geolocation errors are smaller than those of M1, with three-dimensional errors ranging from 0.72 m to 1.60 m.

**Figure 9:**
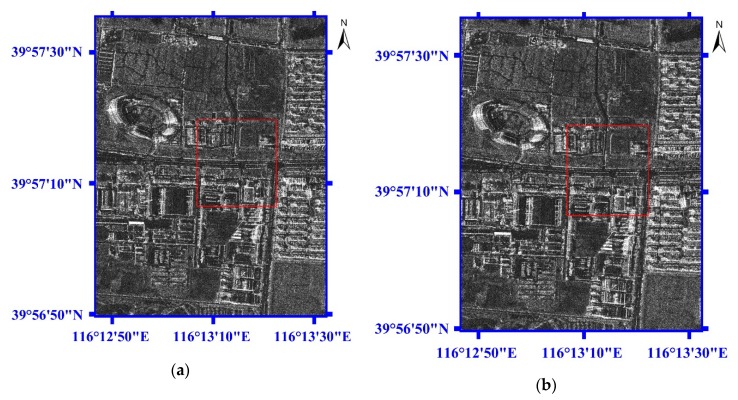

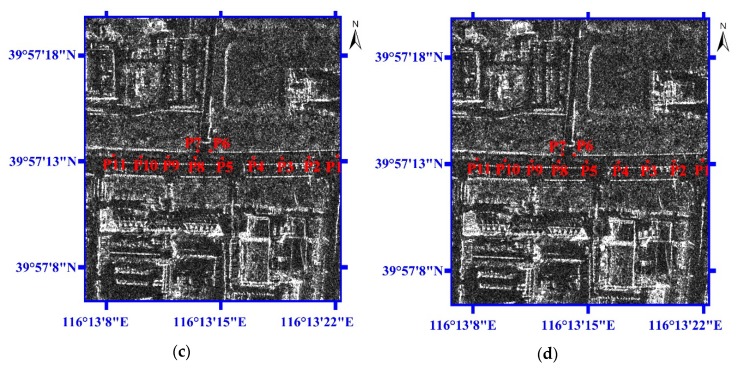
The geolocation results for the street lights and the street barriers located on Xingshikou Road in Beijing. (**a**) The two-dimensional image for M1. (**b**) The two-dimensional image for M2. (**c**) The enlarged image for the red window (Area 2 in Figure 6) in Figure 9a. (**d**) The enlarged image for the red window (Area 2 in Figure 6) in Figure 9b.

Figure 9 shows the geolocation results for the street lights and barriers located on Xingshikou Road in Beijing, taken from Figure 6. The image size of Figure 9 is 2048 × 2048 pixels. Figure 9a,b are the two-dimensional images for M1 and M2, respectively. The red windows in Figure 9a,b are enlarged, as shown in Figure 9c,d, respectively. So, the eleven check points for the street lights (P1~P5 and P8~P11) and the street barriers (P6, P7) located on Xingshikou Road in Beijing can be clearly seen. The red windows in Figure 9a,b have the same longitude and latitude ranges. Compared with Figure 9c, the eleven check points for the street lights (P1~P5 and P8~P11) and the street barriers (P6, P7) located on Xingshikou Road in Figure 9d have obviously shifted longitudinally by several pixels.

**Figure 10:**
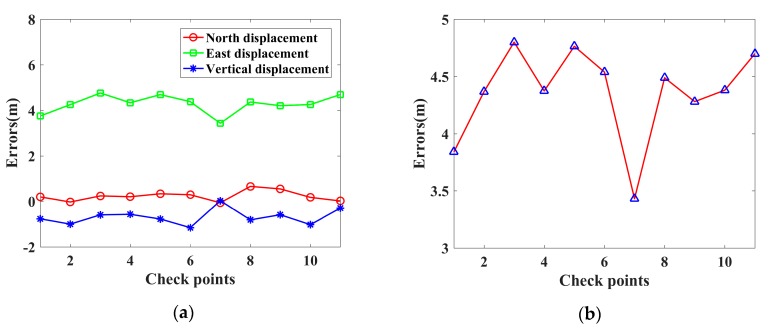

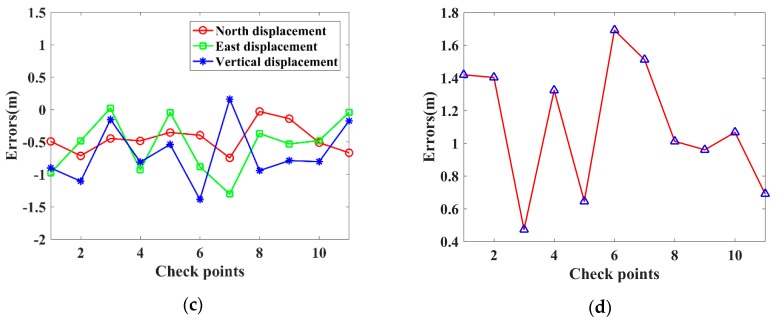
The geolocation errors for the street lights and street barriers located on Xingshikou Road in Beijing with two methods. (**a**,**c**) are the geolocation errors in the north, east and vertical displacements for M1 and M2, respectively. (**b**,**d**) are the three-dimensional errors for M1 and M2, respectively.

Similar to Figure 8, Figure 10 shows the geolocation errors for the street lights and the street barriers located on Xingshikou Road with the two methods mentioned above. Figure 10a,c illustrates the geolocation errors in the north, east, and vertical displacements for M1 and M2, respectively. The red line with circle markers represents the north displacement, the green line with rectangle markers represents the east displacement, and the blue line with asterisks represents the vertical displacement. In Figure 10b,d, the red lines with blue triangle markers represent three-dimensional errors for M1 and M2, respectively.

Table 3 presents the geolocation accuracy for the street lights and street barriers. The QX positioning results are taken as the true positions for the eleven check points. By comparing the geolocation results of M1 (or M2) with QX, we can get the geolocation accuracy of the check points. As shown in Table 3, there are large displacements in east for M1, which are mainly due to atmospheric propagation delay in range. The displacements of the eight corner points in northing (ΔN), easting (ΔE), orthometric height (ΔH), and spatial (Δ**Spatial**) are summarized in Table 3. The M1 geolocation errors are very large; the three-dimensional errors range from 3.43 m to 4.80 m. The M2 geolocation errors are smaller than those of M1, with three-dimensional errors ranging from 0.47 m to 1.69 m.

### 4.2. Computational Efficiency and Accuracy

The computing platform used was a Thinkpad E570 (Lenovo group co., Ltd., Beijing, China) with Core i5-7200U at 2.5GHz, 8GB memory, solid state drive and running the Windows 7 operating system. In addition, the processing software used was MATLAB R2016b (The MathWorks, Inc., Natick, MA, USA). There are two geolocation experiments: (i) The gridded DSM with 1024 × 1024 pixels, (ii) The gridded DSM with 2048 × 2048 pixels. All the geolocation experiments are tested with the TanDEM-X’s spotlight mode SAR image. The main parameters of the SAR image are shown in Table 1.

The scale and processing time are shown in Table 4 to compare the proposed method (M2) to the traditional three-dimensional, indirect geolocation method. The traditional method needs to iterate different times according to different thresholds. In this experiment, the threshold of time difference is set to 0.0001 s. The proposed method is not an iterative method, so it is not necessary to set a threshold.

It is apparent that the proposed fast, three-dimensional, indirect geolocation method is about 12 times faster than the traditional three-dimensional, indirect geolocation method.

The mean errors of the three-dimensional geolocation for check points are shown in Table 5. The geolocation accuracy of the traditional method is the same as that of the proposed method within the scale of 1024 × 1024 and 2048 × 2048.

## 5. Discussion

This paper proposes a fast, three-dimensional, indirect geolocation method using IAGM and DSM data for spaceborne SAR images. The traditional indirect geolocation method based on DSM or DEM data is computationally complex to obtain the accurate azimuth time and slant range, because several iterations must be executed [8,9,10]. The proposed fast method includes two steps to obtain the accurate azimuth time, while keeping the geolocation accuracy. By utilizing the geometric relation between the four corner points and the target, it is easier to estimate the azimuth time. It has to be mentioned that the proposed method can be widely used in any kind of terrain including flat ground, hills, mountains etc. DSM refers to the height of ground surface, buildings, bridges and trees. But DEM contains only elevation information of the terrain, and does not contain other surface information. If there is any need for geolocation regarding buildings, bridges, and other ground surface information, DSM data must be used.

A TanDEM-X SAR image is used to prove this fast, three-dimensional, indirect geolocation method without GCPs. Generally, the geolocation results have no atmospheric propagation delay correction; however, for some special requirements, such as accurate positioning, atmospheric propagation delay correction must be considered. Therefore, a fast, indirect geolocation method is used in such situations. In addition, M1 and M2 have the same computational complexity, and M2 only increases the correction for slant range. The three-dimensional errors in range for the eight corner points at the Banshan Pavilion are 3.76 m to 5.85 m and 0.72 m to 1.60 m for M1 and M2, respectively. For the street lights and barriers located on Xingshikou Road, their three-dimensional errors in range are 3.43 m to 4.80 m and 0.47 m to 1.69 m for M1 and M2, respectively. M1 is a general geolocation method that does not require additional data support. M2 includes atmospheric propagation delay correction, which needs the support of atmospheric data. It has been shown that this fast method can be generally used in spaceborne SAR images for indirect geolocation. It is about 12 times faster than the traditional three-dimensional, indirect geolocation method.

In the pursuit of accurate positioning, future research will consider the solid Earth tides, continental drift, and so on.

## 6. Conclusions

In this paper, a fast, three-dimensional, indirect geolocation method for spaceborne SAR images is proposed. It decreases the calculation time by avoiding several iterations, as used in the traditional indirect geolocating method. For precise geolocation of a SAR image, atmospheric propagation delays have been compensated, and the orthometric height of the DSM data has also been transformed to ellipsoidal height. Therefore, the geolocation could achieve accurate results within 2 m from the true positions for the check points. It has been shown that the proposed method is reliable and efficient for geolocation using TanDEM-X’s spotlight mode SAR images. Moreover, this paper provides a method which can also be used in other spaceborne SAR systems, such as COSMO-SkyMed, Radarsat, Sentinel etc. The accurate azimuth time can be calculated with less than one pulse repetition time (PRT) with very few errors compared to the traditional three-dimensional, indirect geolocation method.

## Figures and Tables

**Table 1:** TanDEM-X’s Parameters.

Parameter Name	Value	Units
Image size	6235×10808	−
Azimuth spacing	0.8705	m
Range spacing	0.4547	m
Slant range to the first pixel	706,315.7084	m
Pulse repetition frequency	3720.4126	Hz
Radar frequency	9649.998153	MHz
The number of orbit state vectors	12	−
Date of acquisition(UTC)	20 December 2018	−
Acquisition mode	Spotlight	−
Producttype	SSC_HS_S	−
Descending/Ascending	Ascending	−
Look direction	Right	−

**Table 2:** The geolocation accuracy for the eight corner points.

Check Points	Δ*N* (m)	Δ*E* (m)	Δ*H* (m)	Δ*Spatial* (m)
P1-M1	1.0942	3.9758	−1.1020	4.2684
P1-M2	0.4080	−0.2308	−0.5400	0.7151
P2-M1	0.2842	4.1319	−1.2079	4.3143
P2-M2	−0.4020	−0.0747	−0.8236	0.9195
P3-M1	−0.0664	3.5610	−1.4886	3.8602
P3-M2	−0.0664	−0.6456	−0.4571	0.7938
P4-M1	0.6374	5.2201	−0.6411	5.2978
P4-M2	0.6374	−0.0382	−0.8710	1.0800
P5-M1	−0.0816	5.3240	−0.2730	5.3316
P5-M2	−0.7678	−0.4600	−1.3277	1.6012
P6-M1	−0.1002	3.6949	−0.6922	3.7605
P6-M2	−0.7864	−0.5117	−0.3195	0.9912
P7-M1	0.0063	4.3063	−0.2809	4.3155
P7-M2	−0.6799	0.0998	−0.2821	0.7429
P8-M1	0.1632	5.7325	1.1457	5.8481
P8-M2	−0.5230	1.0001	0.7717	1.3672

**Table 3:** The geolocation accuracy for the street lights and the street barriers.

Check Points	Δ*N* (m)	Δ*E* (m)	Δ*H* (m)	Δ*Spatial* (m)
P1-M1	0.1944	3.7567	−0.7724	3.8402
P1-M2	−0.4918	−0.9774	−0.9034	1.4189
P2-M1	−0.0275	4.2506	−1.0030	4.3674
P2-M2	−0.7137	−0.4836	−1.1060	1.4023
P3-M1	0.2397	4.7571	−0.5951	4.8002
P3-M2	−0.4465	0.0229	−0.1541	0.4729
P4-M1	0.2034	4.3313	−0.5728	4.3737
P4-M2	−0.4828	−0.9289	−0.8107	1.3240
P5-M1	0.3324	4.6884	−0.7787	4.7642
P5-M2	−0.3538	−0.0458	−0.5379	0.6455
P6-M1	0.2901	4.3786	−1.1601	4.5390
P6-M2	−0.3961	−0.8816	−1.3881	1.6915
P7-M1	−0.0604	3.4302	0.0244	3.4308
P7-M2	−0.7466	−1.3040	0.1605	1.5111
P8-M1	0.6558	4.3651	−0.8153	4.4888
P8-M2	−0.0304	−0.3691	−0.9421	1.0123
P9-M1	0.5451	4.2038	−0.5918	4.2801
P9-M2	−0.1411	−0.5304	−0.7880	0.9603
P10-M1	0.1747	4.2544	−1.0270	4.3801
P10-M2	−0.5115	−0.4798	−0.8043	1.0671
P11-M1	0.0195	4.6897	−0.2976	4.6992
P11-M2	−0.6667	−0.0445	−0.1763	0.6911

**Table 4:** Computational burden.

SN	Scale	Traditional	Proposed
i	1024 × 1024	59.15 s	4.69 s
ii	2048 × 2048	232.82 s	18.58 s

**Table 5:** The mean errors of the three-dimensional geolocation for check points.

SN	Scale	Traditional	Proposed
i	1024 × 1024	1.026 m	1.026 m
ii	2048 × 2048	1.108 m	1.108 m
